# Measuring Acoustic Roughness of a Longitudinal Railhead Profile Using a Multi-Sensor Integration Technique

**DOI:** 10.3390/s19071610

**Published:** 2019-04-03

**Authors:** Dahae Jeong, Han Shin Choi, Yong Je Choi, Wootae Jeong

**Affiliations:** 1 Deptpartment of Robotics and Virtual Engineering, University of Science & Technology, Daejeon 34113, Korea; dahaejeong1@gmail.com; 2 Deptpartment of Mechanical Engineering, Yonsei University, Seoul 03722, Korea; freeaul@yonsei.ac.kr (H.S.C.); yjchoi@yonsei.ac.kr (Y.J.C.); 3 Transportation Environmental Research, Korea Railroad Research Institute, Uiwang 16105, Korea

**Keywords:** multi-sensor, sensor integration, rolling noise, surface roughness measurement, chord offset synchronization method, mobile platform

## Abstract

It is necessary to measure accurately the rolling noise generated by the friction between wheels and rails in railway transport systems. Although many systems have recently been developed to measure the surface roughness of wheels and rails, there exist large deviations in measurements between each system whose measuring mechanism is based on a single sensor. To correct the structural problems in existing systems, we developed an automatic mobile measurement platform, named the Automatic Rail Checker (ARCer), which measures the acoustic roughness of a longitudinal railhead profile maintaining a constant speed. In addition, a new chord offset synchronization algorithm has been developed. This uses three displacement sensors to improve the measuring accuracy of the acoustic roughness of a longitudinal railhead profile, thereby minimizing the limitations of mobile platform measurement systems and measurement deviation. We then verified the accuracy of the measurement system and the algorithm through field tests on rails with different surface wear conditions.

## 1. Introduction

The noise and vibration generated by railway transport systems negatively affect the living environment of nearby residents, whilst the resulting structural defects increase the cost of maintenance.

**Figure 1:**
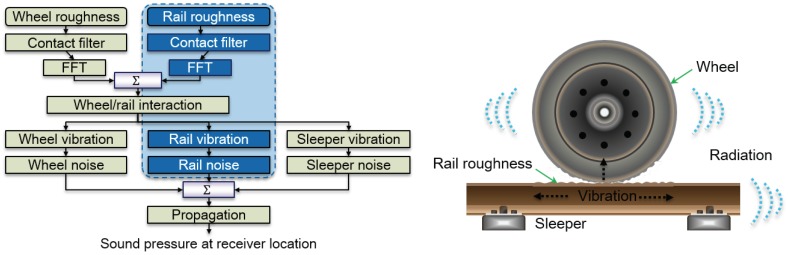
Model of rolling noise generation.

**Figure 2:**
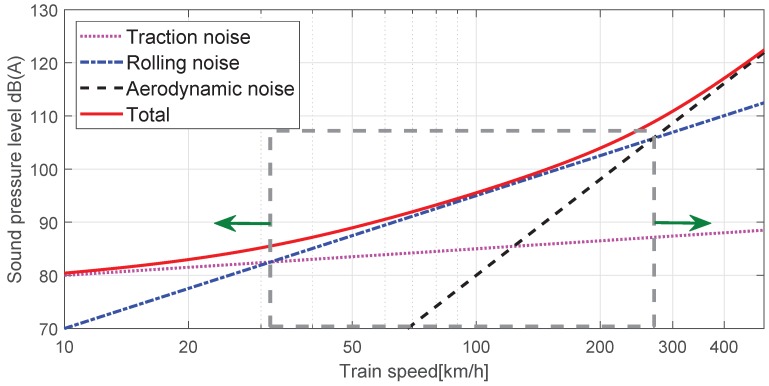
Sound pressure level by train speed.

Although there are composite causes of and propagation paths for railway noise, it is generally classified into traction noise, rolling noise, and aerodynamic noise. Traction noise is generated by a power source such as an engine, propulsion motor and gear system in a car, and equipment installed below the car body, while aerodynamic noise is generated by friction of the front head, body, pantograph, and space between cars with air during high-speed operation. Rolling noise is caused by the interaction between an irregular wheel profile and an uneven rail surface, as shown in the TWINS model in Figure 1 [1,2]. The level of noise during the railway vehicle’s operation depends on the condition of the railway track, the track gradient, the type of vehicle, and the speed. As shown in Figure 2, the impact of traction noise is greatest at low speeds of less than 20 km/h, while that of rolling noise is greatest at speeds ranging between 20 km/h and 250 km/h, with the impact of aerodynamic noise becoming greatest at higher speeds. As technology has reduced the domain of traction noise and aerodynamic noise, the impact of rolling noise has increased over a wider speed range.

Therefore, measurement, prediction, and analysis of rolling noise caused by contact surface irregularities between wheel and rail have become important in railway noise management and track maintenance according to train operation.

The roughness of a rail surface is expressed as a function of the wavelength of the rail, and each wavelength is related to the noise generating frequency according to the speed [3,4]. The wavelength of acoustic roughness related to rolling noise becomes longer as vehicle speed increases, while longer wavelength irregularities on the rail’s surface cause significant dynamic stress on the rail itself and are then transmitted to structure-borne noise, which is an important cause of fastener deformation [5]. Irregularity of a rail surface with a relatively short wavelength is generally defined as the acoustic roughness of the rail and becomes the important cause of rolling noise transmitted into the air with high energy in a high frequency domain. Although it is difficult to distinguish clearly between structure-borne and airborne frequency bands in rolling noise, structure-borne noise generally occurs in a frequency range of 0∼100 Hz, while airborne noise occurs in a frequency range of 30 Hz–5 kHz. At vehicle operating speeds of 50∼120 km/h, the frequency band is 500∼2000 Hz, which is the main interest of this paper. This corresponds to a wavelength of 7∼70 mm and an acoustic roughness of the rail of about 0.1∼100 μm. These values match the measurements of field tracks, as the key frequency band of rolling noise sources was measured at 500∼4000 Hz, while the wavelength of key acoustic roughness ranged as 5∼250 mm. As such, European and international standards specify the wavelength range for measurement and analysis of rolling noise to be 3.15∼630 mm (EN ISO 3095) [6], 3.15∼250 mm (EN 15610) [7], and 10∼1000 mm (EN 13231-3) [8].

A trolley-type mobile device is generally used to analyze rolling noise by measuring the acoustic roughness of a rail surface. Although an accelerator sensor installed in the mobile platform [9] or a displacement sensor fixed on the platform [10] is commonly used to measure rail surface roughness, a laser displacement sensor or other non-contact sensor can also be used. A displacement sensor measures relative displacement from the top platform to the rail surface. The sensor in contact with the rail surface measures the roughness of the latter with a variation of the displacement value caused by the rugged surface when the platform of the measuring apparatus travels at a constant height from the rail surface. However, the rail roughness value of an operating line becomes an irregular surface shape with a wide frequency band since corrugations, ruggedness, and cracks of varying sizes are generated by the train operating at different speeds and friction with the wheel. Therefore, the reference system of the instrument that measures such a surface varies according to the pitching motion in which the front and rear wheel contact points of the apparatus shake up and down with respect to the longitudinal rail direction. Such a moving platform does not have a fixed reference point for measurement, and the amplitude and phase of the measured value become inaccurate as the sensor tilts.

The authors of this paper designed an automatic measurement platform that corrects the structural problems in recent mobile measurement systems with built-in displacement sensors and propose a new algorithm to reduce the deviation of measured values while improving the accuracy of measurement of rail surface acoustic roughness. We then verified the accuracy of the algorithm through a rail track field test.

## 2. Measurement and Analysis of Rail Acoustic Roughness

### 2.1. Measurement of Acoustic Roughness Using an Acceleration Sensor

Measuring a longitudinal railhead profile with an accelerator sensor [9] involves a fixed acceleration sensor on a rigid body inside a trolley-type device and calculating the acoustic roughness of the rail surface by a double integral of measured values. Compared to displacement sensor-based systems, such accelerometer-based measuring systems are not sensitive to errors from the pitching motion and shaking of the displacement sensor measuring systems, since they do not require rail reference data. However, they are sensitive to the vibration of the mobile measurement system itself, and the acceleration sensor needs to be dynamically isolated from the measurement system. Moreover, it is essential to slow down measurement speed and maintain constant speed movement within the measurement time to maintain contact between the acceleration sensor and the rugged rail surface while the measuring system is moving. It is also difficult to verify the accuracy of the acoustic roughness displacement value estimated by a double integral of the acceleration value. Because of such drawbacks, the mobile measurement systems that use a displacement sensor have recently become more popular.

### 2.2. Measurement of Acoustic Roughness Using a Displacement Sensor

**Figure 3:**
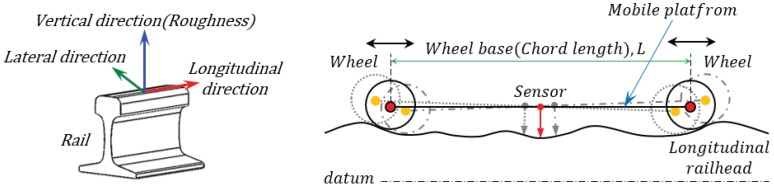
Mobile platform to measure longitudinal railhead profile.

Although displacement sensor-based measuring systems can measure surface roughness directly from the rail surface condition without additional calculations such as integrals, while the measuring system is in motion, the sensor, fixed to a device that pitches while in motion, measures only relative displacement and thus becomes the main cause of measurement error since it is difficult to set the measurement reference line (datum) of the rail, as shown in Figure 3.

To compensate for such errors, the chord-based method [11] assumes rail surface roughness to be a sinusoidal wave (y(x)=Ysin(kx)=Ysin(2πx/λ)) with amplitude *Y* and wavelength λ when using a measurement sensor, and calculates the transfer function of the rail roughness value and the measured value by using the ratio of the chord length (*L*) and the length from the device edge to the sensor position. However, the error-compensation accuracy is limited because there exists an unmeasurable or amplified wavelength domain depending on the ratio (position) of the sensor arrangement. Therefore, the chord offset method, mainly used to analyze track curvature, is applied to a small mobile measuring system using multiple sensors to analyze rail acoustic roughness and therefore reduce the limitations of using only one sensor [12]. Although the chord offset method—using multiple sensors—offers the ability to analyze measured data in a sinusoidal wave profile such as track curvature calculation, wavelength compensation is limited according to the distance between sensors. Moreover, accuracy greatly deteriorates when compensating a railhead profile that includes cracks and ruggedness that do not have a sinusoidal wave profile, and it is also difficult to compensate disturbances if additional noise sources exist such as eccentricities of the driving wheel.

Therefore, the existing methods that analyze rail surface irregularity using a displacement sensor are adequate for measuring rail surface roughness with longer wavelengths than the length of the measuring system (wheelbase) but may not be suitable for shorter rail wavelengths, which significantly affect acoustic roughness, since the unmeasurable or amplified wavelength band increases. Particularly, considering that rolling noise has wavelengths of λ = 5∼250 mm, a method of analysis that considers the measured wavelength range to analyze the rail acoustic roughness accurately is needed.

Another cause of measurement errors by trolley-type measurement systems with a single displacement sensor is the impact of periodic disturbances on measurement such as driving wheel eccentricity. It is difficult to compensate for errors caused by wheel rotation or eccentricity in a single sensor-based system, but adequate compensation is critical for accurate analysis of the results.

Methods to reduce the distortion of data by a single-sensor mobile measurement system include using a sliding motion point contact method instead of a point contact method involving the driving wheel mounted on the platform [13], but it is difficult to automate such a system, and it can generate significant deviation in the results depending on the person measuring. Another way to overcome the limitations of a single-sensor-based device is to use a large platform length, wheelbase (*L*), or driving wheel diameter, but the result would also have physical limitations in terms of manufacturability or equipment mobility for field measurement.

Therefore, to compensate for errors in measured values and minimize the offset or amplification of measured wavelengths, we propose an algorithm with multiple sensors for mobile measurement systems that have a designated wheel size and wheelbase, or chord length (*L*), considering manufacturability and equipment mobility. The new chord offset synchronizing method presented in the next section increases data accuracy and reduces the effect of disturbances from the driving wheel at the same time by compensating the amplitude and phase of measured values based on at least three sensors in the structure of a manufactured mobile measurement system.

## 3. Chord Offset Synchronizing Method

### 3.1. Single Sensor Measurement Compensation

**Figure 4:**
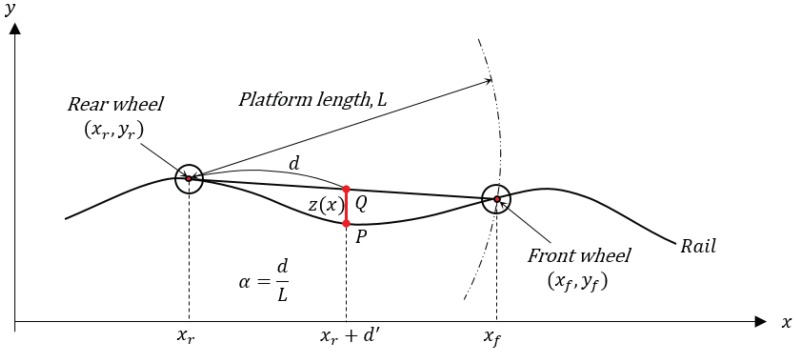
Single-sensor-based chord offset synchronizing model (*d* is the distance between $x_{r}$ and the sensor, and $d^{\prime }$ is the projection of *d*).

As depicted in Figure 4, the algorithm assumes that a measurement system with platform length (or chord length) of *L* performs a pitching motion with a longitudinal tilt on the rail with a sinusoidal wave profile of (y(x)=Ysin(kx)=Ysin(2πx/λ)) with an arbitrary wavelength [11]. In the model, we also assume that the tilted slope from two center points of the wheels is consistent with the slope from the contact points of the front and rear wheel, and the *x* coordinates of the center point and contact point of the wheels are the same. Define the contact point of the rear wheel of the platform and the rail to be $\left(x_{r},y_{r}\right)$ and the contact point of the front wheel to be $\left(x_{f},y_{f}\right)$. Considering a circle equation with the rear wheel as the center of the circle, we can obtain the front wheel contact point $\left(x_{f},y_{f}\right)$ of the inclined measurement platform and calculate the relative measurement value of the displacement sensor fixed to an arbitrary position as follows. Here, the errors generated by the incline of the sensor fixed to the platform and by the vibration are assumed to be very small—and therefore ignored—compared to the error generated by the pitching motion of the platform body.

This assumes that the system measures while moving in the positive *x*-axis direction on the coordinate $\left(x_{f}>x_{r}\right)$ and that the driving wheel moves while in contact with the rail surface with a sinusoidal wave profile, and thus, the coordinates of the contact of two wheels with the rail can be expressed in Equations (1) and (2)

(1) $$y_{r}=Ysin\left(kx_{r}\right)$$

(2) $$y_{f}=Ysin\left(kx_{f}\right)$$

where *Y* refers to the amplitude of the sinusoidal wave rail, while *k* is 2π/λ. Equation (3) is a circle equation with the rear wheel as the center and the radius of *L* while the system is inclined.

(3) $$L^{2}={\left(x_{f}-x_{r}\right)}^{2}+{\left(y_{f}-y_{r}\right)}^{2}$$

We can solve the three simultaneous equations through a simulation to obtain the cross-point of the sinusoidal wave rail and the circle, i.e., the coordinates $\left(x_{f},y_{f}\right)$ of the front wheel.

Therefore, the sensor position on the rail (*P* in Figure 4) is:

(4) $$P=y(\left(1-\alpha \right)y_{r}+\alpha y_{f})$$

From the *x* coordinate of the sensor,

(5) $$x_{r}+d^{\prime }=\left(1-\alpha \right)x_{r}+\alpha x_{f},$$

we can calculate the sensor position on the platform (*Q* in Figure 4).

(6) $$Q=\left(1-\alpha \right)y\left(x_{r}\right)+\alpha y\left(x_{f}\right)$$

Since the measured value (z(x)) of the sensor is the distance from the sensor position on the platform to the rail waveform on a vertical line, it can be summarized as follows.

(7) $$\begin{array}{cc} z(x) & =P-Q\\ & =y\left(\left(1-\alpha \right)x_{r}+\alpha x_{f}\right)-\left\{\left(1-\alpha \right)y\left(x_{r}\right)+\alpha y\left(x_{f}\right)\right\}\\ & =Ysin\left(k\left(1-\alpha \right)x_{r}+\alpha kx_{r}\right)-\left(1-\alpha \right)Ysin\left(kx_{r}\right)-\alpha Ysin\left(kx_{f}\right) \end{array}$$

where $\alpha =d/L=d^{\prime }/\left(x_{f}-x_{r}\right)$

Thus, we can calculate the amplitude $\left(Z=\left|z(x)\right|\right)$ and phase (Φ=phase(z(x))) of the value measured by the sensor from an arbitrary sensor position on the platform. Furthermore, the transfer function of the measured value of the rail surface can be defined as follows.

(8) H(λ)=Z(λ)/Y(λ)

**Figure 5:**
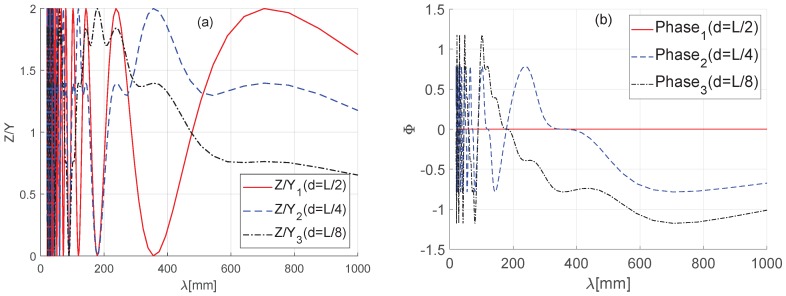
(**a**) Magnitude of the transfer function. (**b**) Phase angle of the transfer function.

Figure 5 depicts the amplitude and phase angle of the transfer function calculated with Equation (7).

Here, the wheelbase (*L*) of the platform is considered as a constant value. As a result of our analysis, the graph of the amplitude and phase angle of the transfer function changes according to the position (*d*) of the measurement sensor and the wavelength domain of the rail. Assuming the sensor is positioned at the center of the platform (*d* = *L*/2), the amplitude of the transfer function is amplified twice when the wavelength λ of the rail surface becomes L/(2n−1)-times the wheelbase. In addition, when the wavelength λ of the rail surface is L/2n-times the wheelbase, the amplitude of the transfer function is close to zero, and the wavelength of the rail surface cannot be measured.

Although there is no difference in the phase of the transfer function and the measured value if the sensor is positioned at the center of the platform (*d* = *L*/2), the phase angle of the observed transfer function changes when the position of the sensor changes in other cases.

As a result, when using a single sensor to measure rail surface roughness, the measured value differs according to the wavelength (λ) of the rail surface, the length (*L*) of the system, and the sensor position (*d*), even if we analyze the measured value by reflecting the platform incline, making it difficult to compensate for error in values measured by the existing trolley-type mobile systems. Therefore, it is necessary to reduce these errors by offsetting the amplification or attenuation of measured values through the design of the platform length (*L*) in consideration of the physical size of the platform and using the position of multiple sensors by considering the wavelength range that generates rolling noise.

### 3.2. Analysis of Acoustic Roughness by a Convergence of Multiple Sensors

This section describes chord offset synchronization, whereby results can be obtained by compensating the measured values of multiple sensors fixed at different positions to reduce errors in measured values more than those produced by single-sensor measurement systems. Multi-sensor-based chord offset synchronization analyzes the response of the measured rail surface values to obtain the relative positions of the sensors on the platform, so the amplitude and phase angle of the transfer function are not amplified or attenuated and synchronize the measured values to find optimal measurement results. This method results in values differently compensated according to the relative distance between sensors and can produce more accurate measured values on a wide rail surface wavelength spectrum than conventional analytical methods. It can also easily discriminate between cracks and rail corrugations in any analysis. The measured values need to be synchronized in the same position to converge the sensors used in different positions, and at least three sensor values are needed to increase the accuracy of the synchronized results. Moreover, the convergence of the measured values from multiple sensors can reduce the impact of disturbances that affect the measured values by incorporating the difference in sensor measurement times while the mobile platform is moving. In our research, we calculated the optimal sensor arrangement for frequency analysis of the rolling noise section through sensor interval setting and calculating the position of the center sensor to determine the optimal positions of at least three sensors to be fastened to the platform for compensation of rail acoustic roughness.

**Figure 6:**
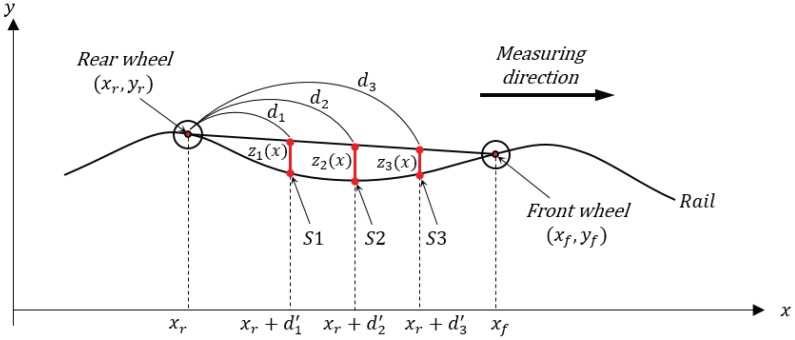
Multi-sensor-based chord offset synchronizing model ($\alpha_{i}=d_{i}/L$, and $d_{i}$ is the distance from $x_{r}$ to the *i*th sensor (*i* = 1, 2, 3)).

First, we modeled the multi-sensor-based chord offset synchronization using three sensors to calculate the measured value and phase difference of each sensor, as depicted in Figure 6. The three sensors were designated as S1, S2, and S3 in order starting with the front sensor, while the sensor position ratios to the inclined platform $\left(L^{\prime }=x_{f}-x_{r}\right)$ were defined as $\alpha_{1},\alpha_{2},\alpha_{3}$, respectively. We then calculated the contact point with the driving wheel in consideration of the incline of the railhead in the sinusoidal wave profile and the system and calculated the sensor measurement according to the position ratio.

Since the measured value is the distance from the contact point between sensor and platform, the measured value $z_{i}\left(x\right)$ of each sensor can be expressed as follows.

(9) $$\begin{array}{cc} z_{i}\left(x\right) & =P_{i}-Q_{i}\\ & =y\left(\left(1-\alpha_{i}\right)x_{r}+\alpha_{i}x_{f}\right)-\left\{\left(1-\alpha_{i}\right)y\left(x_{r}\right)+\alpha_{i}y\left(x_{f}\right)\right\}\\ & =Ysin\left(k\left(1-\alpha_{i}\right)x_{r}+\alpha_{i}kx_{r}\right)-\left(1-\alpha_{i}\right)Ysin\left(kx_{r}\right)-\alpha_{i}Ysin\left(kx_{f}\right)\\ & \left(i=1,2,3\right) \end{array}$$

Therefore, we can obtain the amplitude and phase angle output by each sensor position $(\alpha_{1},\alpha_{2},$ and $\alpha_{3})$ on the platform in the acoustic roughness wavelength range measured by the three sensors. The platform length or chord length (*L*) is assumed to be constant for the calculation.

**Figure 7:**
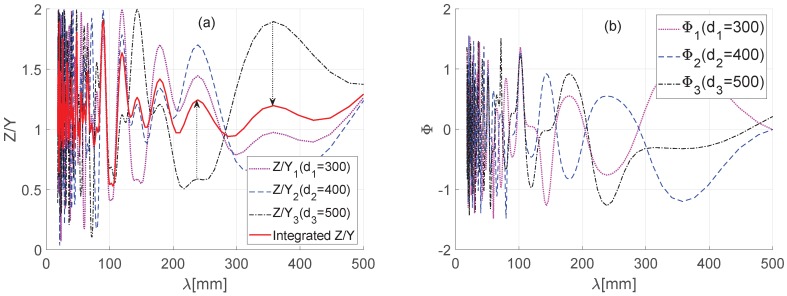
(**a**) Magnitude of the transfer function; (**b**) Phase angle of the transfer function.

To study the correlation between railhead and platform length, we set the important wavelength range of surface roughness to 0∼500 mm and calculated the amplitude (*Z*) and phase angle Φ of the values measured by the sensors. The results shown in Figure 7 indicate that the transfer function of the wavelength of the value measured on an arbitrary sensor position $(d_{1}=300,d_{2}=400,$ and $d_{3}=500\;\mathrm{mm})$ is amplified or attenuated to 0∼2-times, while the phase angle has an error of −π/2∼π/2. Therefore, we can assume the value measured in each sensor is a sinusoidal wave of different amplitude and phase angle as shown in Equation (10) below.

(10) $$z\left(x\right)=Zsin\left(\frac{2\pi x}{\lambda }+\Phi \right),\left(i=1,2,3\right)$$

where the measurement position of each sensor can be converged through a mutual compensation, as shown in Equation (11).

(11) $$z^{*}\left(x\right)=f\left\{z_{1}\left(x\right),z_{2}\left(x\right),z_{3}\left(x\right)\right\}$$

(12) $$\begin{array}{c} Z^{*}=\frac{1}{3}\left|Z_{1}sin\left(\left(\frac{2\pi }{\lambda }\right)x+\Phi_{1}\right)+Z_{2}sin\left(\left(\frac{2\pi }{\lambda }\right)x+\Phi_{2}\right)+Z_{3}sin\left(\left(\frac{2\pi }{\lambda }\right)x+\Phi_{3}\right)\right| \end{array}$$

Figure 7 shows the measured values from the three sensors and the converged result value. The results indicate that the converged value has a lower magnitude of amplification or attenuation than the individual sensor values.

## 4. A Mobile System to Measure Rail Acoustic Roughness

### 4.1. Design of the Mobile Measurement System with Three Sensors

**Figure 8:**
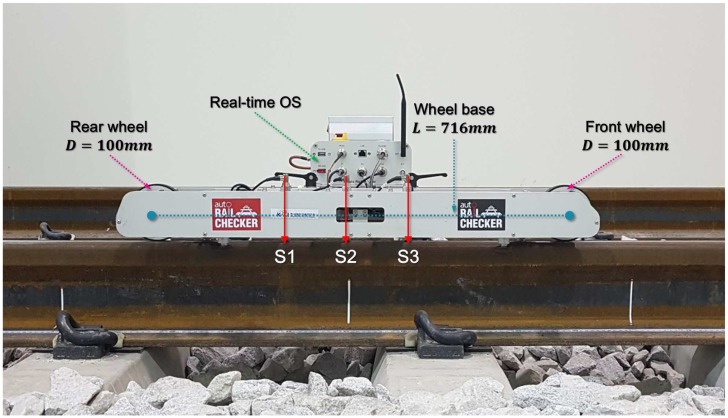
Auto Rail Checker (ARCer) for measuring the longitudinal railhead profile.

It is difficult to maintain constant speed in the trolley-type system during measurement of railhead acoustic roughness since it is a manual system requiring a person to push the trolley device. However, maintaining constant speed with the measurement system is essential during measurement since acoustic roughness is particularly sensitive to changes in measurement speed. To reflect this, we manufactured an automated measurement system called the Auto Rail Checker (ARCer), shown in Figure 8, to measure the acoustic roughness of a rail surface at a constant controlled speed. This newly-developed ARCer consists of a real-time operating system, a speed controller, a rotary encoder to measure distance, three displacement sensors with adjustable positions, and wireless data communication. The ARCer has a wheelbase of 716 mm and front and rear driving wheels of 100 mm in diameter. It can move at a maximum speed of 4 m/s, and the measuring speed is normally 0.5 m/s. The measurement and analysis of the longitudinal railhead roughness profile are based on EN 15610:2009 [7].

The system uses three LVDTs (Linear Variable Differential Transformers, Transducers) with a resolution of 0.1 μm. The tip of each sensor has a ceramic ball of 10 mm in diameter to insulate electrical signals and noise from the rail. A spring was added to the sensor body to minimize the impact during movement at speed and to improve contact force between the rail surface and sensor to allow stable measurement of rail cracks and welds.

### 4.2. Sensor Position to Minimize the Impact of Driving Wheels

**Figure 9:**
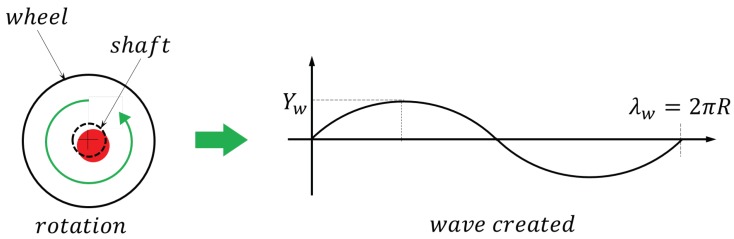
Periodic wheel noise generation.

Sensor position and measurement speed significantly affect measurement precision when measuring acoustic roughness with ARCer. A mobile measurement platform such as ARCer, in particular, can cause disturbances with a circumferential period (λ=πD) due to wheel eccentricity, machining of the drive wheel bearing, and assembly instability as the driving wheel rotates, as shown in Figure 9.

**Figure 10:**
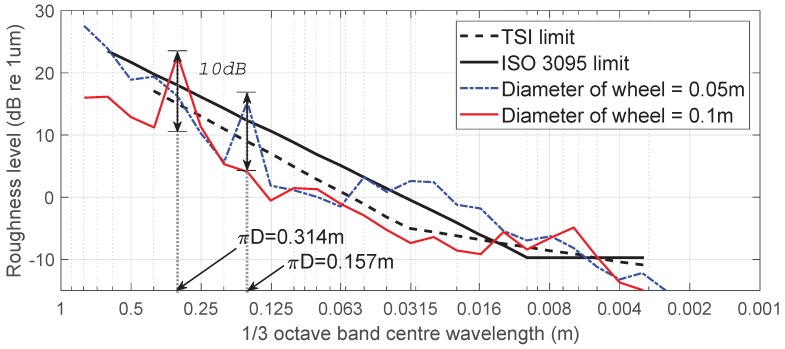
Amplified roughness level according to wheel diameter (TSI: Technical Specification for Interoperability).

The generation of disturbances related to wheel rotation was confirmed through analysis of the octave band spectrum during ARCer field testing. Figure 10 shows the measurement results from a rail surface using the driving wheels of different diameters in the same system. The roughness level is expressed in decibels, given by the following equation:

(13) $$L_{r}=10\cdot \log\left(r^{2}/r_{o}^{2}\right)$$

where $L_{r}$ is the acoustic roughness level in dB, *r* is the root mean square roughness in μm, and $r_{0}$ is the reference roughness, 1 μm [7]. The measurement using a driving wheel diameter of 50 mm and 100 mm had a direct impact on acoustic roughness at a wavelength of λ=πD = 0.157 m and λ=πD = 0.314 m, respectively, and increased the level of acoustic roughness by 10 dB each, as shown in Figure 10.

**Figure 11:**
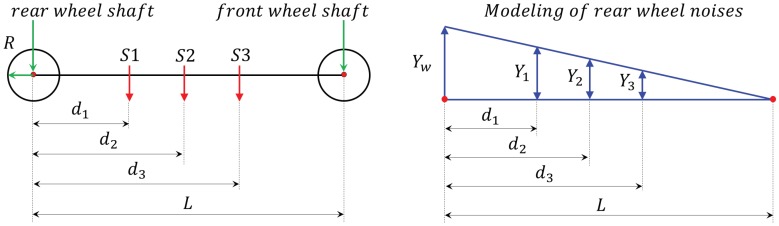
Models of wheel noise.

Therefore, we assumed the disturbance generated by the driving wheel to be a sinusoidal wave having a period of λ=πD and an amplitude of $Y_{w}$ and modeled the disturbance mechanism generated by the system as shown in Figure 11. Firstly, we assumed that the magnitude of a disturbance generated by a driving wheel was inversely proportional to the distance between the axis of that driving wheel and the sensor and defined the magnitude of the sensor disturbances generated by the rear wheel to be $Y_{1},Y_{2},$ and $Y_{3},$ respectively, as shown in Figure 11.

Next, assuming the wheelbase or platform length (distance between front and rear wheels) to be *L*, the radius of the wheel to be *R*, and the distance from the rear wheel to each of the sensors to be $d_{1},d_{2},$ and $d_{3},$ respectively, the sensor disturbances by the disturbance $\left(Y_{w}\right)$ generated by the rear wheel can be expressed as follows.

(14) $$Y_{1}=Y_{w}\left(1-\frac{d_{1}}{L}\right)sin\left(\frac{2\pi x}{\lambda_{w}}\right)$$

(15) $$Y_{2}=Y_{w}\left(1-\frac{d_{2}}{L}\right)sin\left(\frac{2\pi x}{\lambda_{w}}-\frac{2\pi (d_{2}-d_{1})}{\lambda_{w}}\right)$$

(16) $$Y_{3}=Y_{w}\left(1-\frac{d_{3}}{L}\right)sin\left(\frac{2\pi x}{\lambda_{w}}-\frac{2\pi (d_{3}-d_{1})}{\lambda_{w}}\right)$$

Moreover, we can converge the independently-generated sensor disturbances with the multi-sensor chord offset method [12] as shown below to calculate the integrated waveform $\left(Y_{f}\right)$. The convergence method averages sensor values that are mutually independent and are different in phase to generate a new waveform, as shown in Equation (17), and calculates the relative distance between sensors to minimize the amplification and attenuation of the generated value to arrange sensor positions.

(17) $$\begin{array}{cc} Y_{f} & =\frac{1}{3}\left(Y_{1}+Y_{2}+Y_{3}\right)\\ & =\frac{Y_{w}}{3}\{\left(1-\frac{d_{1}}{L}\right)sin\left(\frac{x}{R}\right)+\left(1-\frac{d_{2}}{L}\right)sin\left(\frac{x-(d_{2}-d_{1})}{R}\right)\\ & \;+\left(1-\frac{d_{3}}{L}\right)sin\left(\frac{x-(d_{3}-d_{1})}{R}\right)\}\\ & =\frac{Y_{w}}{3}sin\left(\frac{x}{R}\right)\left\{\left(1-\frac{d_{1}}{L}\right)+\left(1-\frac{d_{2}}{L}\right)cos\left(\frac{d_{2}-d_{1}}{R}\right)+\left(1-\frac{d_{3}}{L}\right)cos\left(\frac{d_{3}-d_{1}}{R}\right)\right\}\\ & \;-\frac{Y_{w}}{3}cos\left(\frac{x}{R}\right)\left\{\left(1-\frac{d_{2}}{L}\right)sin\left(\frac{d_{2}-d_{1}}{R}\right)+\left(1-\frac{d_{3}}{L}\right)sin\left(\frac{d_{3}-d_{1}}{R}\right)\right\} \end{array}$$

**Figure 12:**
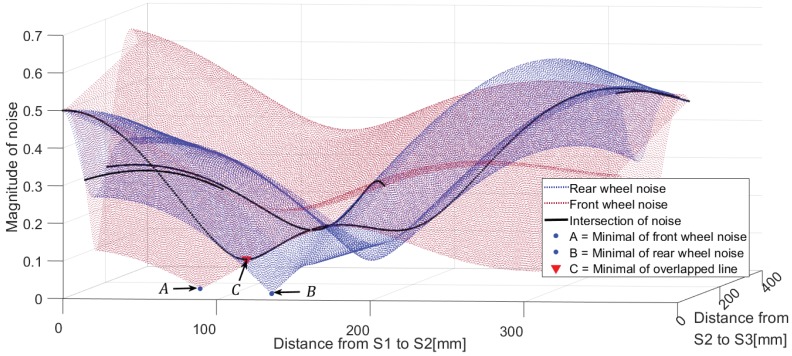
Magnitude of wheel noise.

We calculated the magnitude $\left(Y_{f}\right)$ of the disturbance related to wheel rotation by varying the distance from the reference sensor (S2) on the platform to the front and rear sensors to check the response of the integrated measurement value. The impact of the disturbance from the front wheel can be modeled and the magnitude can be calculated in the same way. Figure 12 shows the calculation of the magnitude of disturbance generated by the front and rear wheels after varying the distance from the reference sensor. The relative distance of the sensor that minimizes the disturbance response calculated for the front and rear driving wheels of 100 mm in diameter becomes Point A for the rear wheel and Point B for the front wheel. Although A and B can attenuate the magnitude $\left(Y_{w}\right)$ of the disturbance, the position that can minimize this magnitude by both driving wheels is Point C. Figure 12 confirms that for the position of Point C, the disturbance response can be minimized when the three sensors are arranged at intervals of about 105 mm (≈πD/3) from the reference sensor (S2).

### 4.3. Sensor Position to Improve Measurement Accuracy

**Figure 13:**
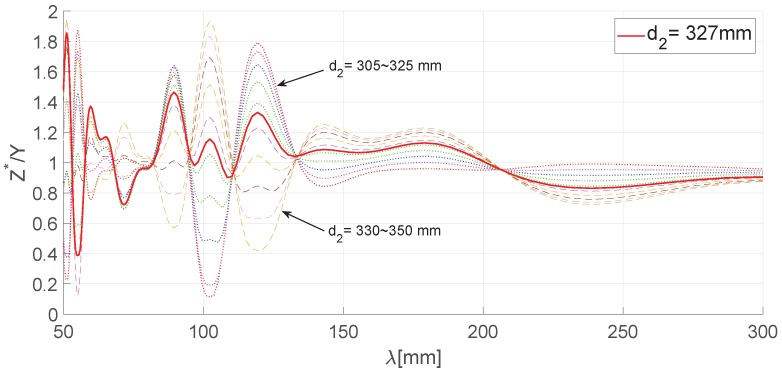
Optimal point algorithmic result.

We calculated magnitude (*Z*) and phase (Φ) corresponding to the measured value of each sensor by varying the distance $\left(d_{2}\right)$ from the rear wheel to the reference sensor to obtain the position (S2) of the reference sensor using the relative position values $\left(|d_{2}-d_{1}|,|d_{2}-d_{3}|\right)$ between the sensors to minimize the disturbance generated by the wheels of the platform and compared them with the mutually-compensated results from multi-sensor-based chord offset synchronization. The graph in Figure 13 shows the magnitude ratio of the compensated measurement values when varying the position $\left(d_{2}\right)$ of the reference sensor on the platform from the center point (*L*/2 = 358 mm) of the system. To find the position for the reference sensor so that the minimum magnitude of the response curve would be obtained, we assumed $Z^{*}$ to be the compensated measurement value using the convergence algorithm and selected the response curve that minimizes the response to rail surface roughness from among $Z^{*}$. We used the function that minimizes the magnitude average for the whole rail wavelength section to find the minimum response curve.

As depicted in Figure 13, the position $\left(d_{2}\right)$ of the reference sensor for the optimal response curve can be classed as 327 mm, and the mean $\left(|H{\left(\lambda \right)}^{*}|\right)$ was 1.05 in that case, while other curves showed a higher mean value. The relative positions of the other two sensors from the reference sensor were $\;d_{1}=$ 222 mm and $d_{3}=$ 432 mm.

As a result, it is possible to determine the sensor positions that can minimize measurement value errors by applying the multi-sensor-based chord offset synchronization algorithm to the sensor response compensation integrated with the impact of the wheel-generated disturbances. Although errors can still occur in measurement values of the short wavelength band, these can be resolved through a data filtering process in spike removal and curvature processing [6] during analysis of the one third octave band in the short wavelength.

## 5. Testing and Results

We measured longitudinal rail acoustic roughness on two rail tracks with different rail surface characteristics to verify the performance of the ARCer and the proposed multi-sensor-based chord offset synchronization algorithm.

**Figure 14:**
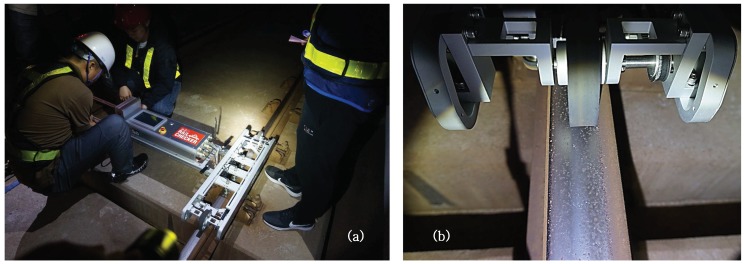
Field measurement of the track A; (**a**) Device setup; (**b**) operating wheel and railhead.

**Figure 15:**
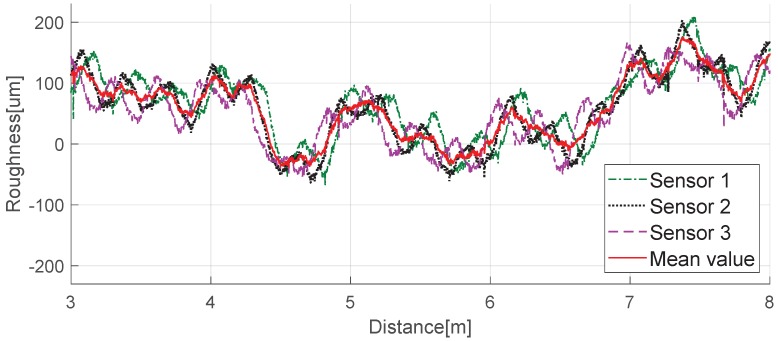
Longitudinal railhead roughness of Track A.

**Figure 16:**
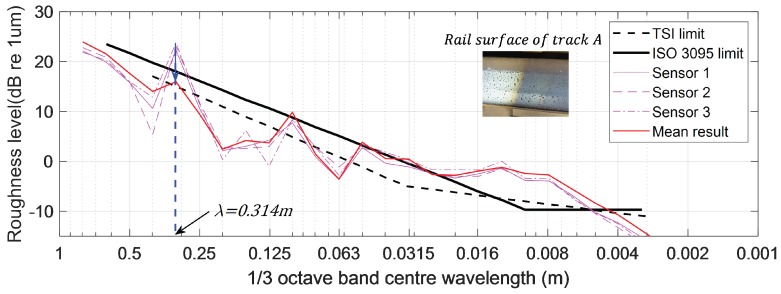
Spectral analysis of Track A railhead roughness.

The selected track, Track A, is a straight section for unmanned trains and is well-maintained with no rail corrugation (see Figure 14). We repeatedly measured a 10-m section of Track A using ARCer on 100 mm-diameter wheels and tested if the measured values minimized the driving wheel disturbance. Figure 15 and Figure 16 show a comparison of the measured acoustic roughness and the railhead acoustic roughness spectrum obtained from spectral analysis before and after applying the algorithm. As shown in the results of spectral analysis in Figure 16, the measured railhead acoustic level of Track A was somewhat high in the short wavelength spectrum. Although the roughness level was high at the wavelength of πD= 0.314 m related to the wheel diameter, which often occurs with a mobile measurement system, phase angle synchronization averaging reduced the impact of wheel rotation on the spectrum by about 10 dB.

**Figure 17:**
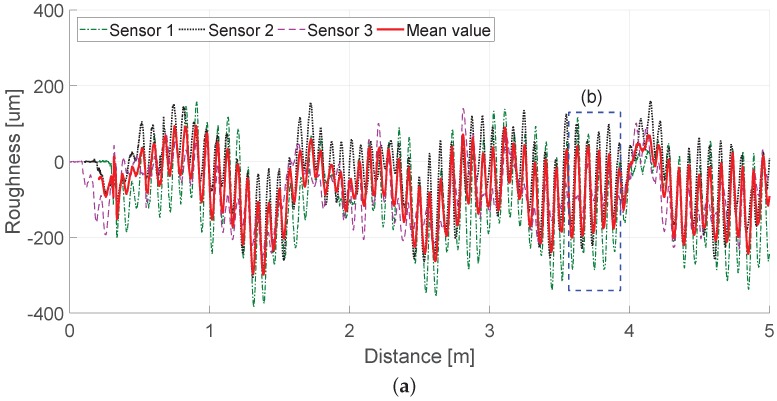

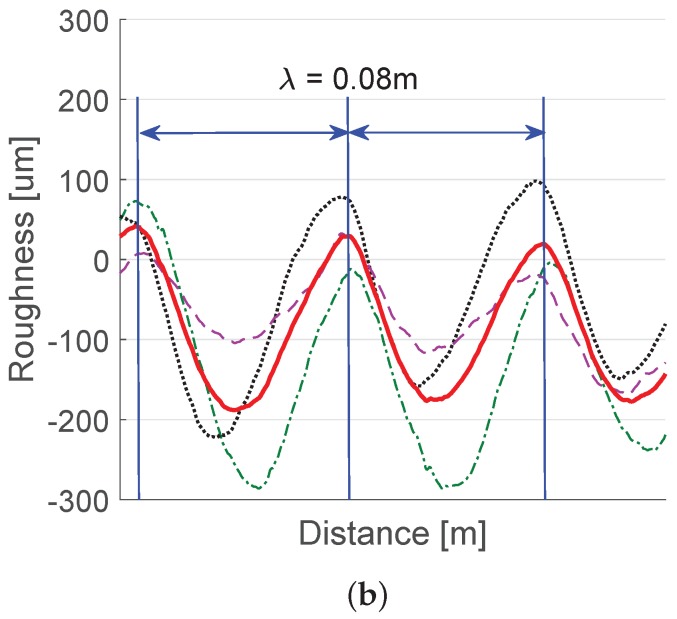
(**a**) Longitudinal railhead roughness of Track B; (**b**) magnified area of the railhead roughness.

**Figure 18:**
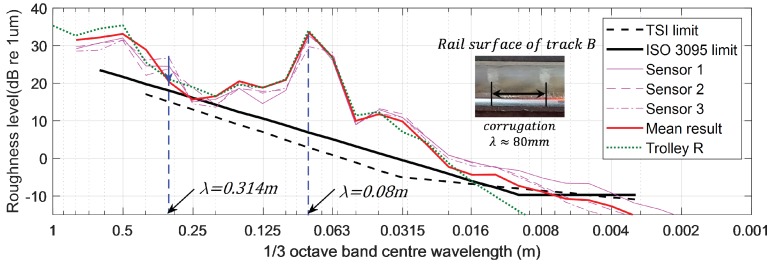
Spectral analysis of the Track B railhead roughness.

Another test was conducted, this time on Track B. Track B, which unlike Track A, has clear corrugations with a wavelength of about 80 mm, and here, we repeatedly measured a 10-m section to verify the proposed multi-sensor-based chord offset synchronization. Three sensors were installed at the optimal positions obtained earlier. Figure 17 and Figure 18 show railhead acoustic roughness and the results of spectrum analysis. As shown in the results of the spectral analysis in Figure 18, the algorithm indicated the condition of corrugation at wavelength $\lambda_{w}=$ 0.08 m accurately and reduced the level of disturbance expected to be generated by the wheel at a wavelength of $\lambda_{w}=$ 0.314 m by about 5 dB. Figure 18 also confirms that the results are very similar to those measured by a commercialized system from Company R, used for measurement of corrugation. The test results on two tracks with different characteristics confirm that the algorithm can minimize the impact of mobile system driving wheels and errors in measurement values in the focused wavelength spectrum of rail acoustic roughness.

## 6. Conclusions

The trolley-type system used to measure railhead acoustic roughness can generate distorted measurements depending on the type of installed sensor, speed, and method of measurement, as well as the way the data are processed. We developed an automatic mobile measurement platform (ARCer) that enabled constant speed control to improve measurement accuracy and a method using a displacement sensor that has recently been widely used. The acoustic roughness levels calculated by existing mobile measurement systems can be affected by the diameter of the driving wheels and sensor position. Therefore, we proposed a chord offset synchronization that involves installing three sensors on a platform and adjusts the relative positions to compensate for disturbances generated by measurement system wheel movement and to reduce errors in measured values. We tested the proposed method on two rail tracks with different characteristics for verification. The results show that analysis of the field measurement values compensated for driving wheel disturbance in the acoustic roughness values by up to 10 dB, with the accuracy of those measured values confirmed by comparing them to measurements by a commercial system of a track with well-developed corrugation. While the proposed chord offset synchronization requires at least three sensors instead of one, it can increase stability over the existing system and is applicable in a variety of ways to mobile platforms.
